# Enantiomer-specific activities of an LRH-1 and SF-1 dual agonist

**DOI:** 10.1038/s41598-020-79251-9

**Published:** 2020-12-17

**Authors:** Suzanne G. Mays, Józef Stec, Xu Liu, Emma H. D’Agostino, Richard J. Whitby, Eric A. Ortlund

**Affiliations:** 1 Department of Biochemistry, Emory University, Atlanta, GA 30322 USA; 2 School of Chemistry, University of Southampton, Southampton, Hants SO17 United Kingdom; 3 Present Address: Centre for Genomic Regulation, Carrer Dr. Aiguader, 88, 08003 Barcelona, Spain; 4 Present Address: Department of Pharmaceutical Sciences, College of Pharmacy, Marshall B. Ketchum University, 2575 Yorba Linda Blvd, Fullerton, CA 82831 USA

**Keywords:** X-ray crystallography, Mechanism of action

## Abstract

Chirality is an important consideration in drug development: it can influence recognition of the intended target, pharmacokinetics, and off-target effects. Here, we investigate how chirality affects the activity and mechanism of action of RJW100, a racemic agonist of the nuclear receptors liver receptor homolog-1 (LRH-1) and steroidogenic factor-1 (SF-1). LRH-1 and SF-1 modulators are highly sought as treatments for metabolic and neoplastic diseases, and RJW100 has one of the few scaffolds shown to activate them. However, enantiomer-specific effects on receptor activation are poorly understood. We show that the enantiomers have similar binding affinities, but RR-RJW100 stabilizes both receptors and is 46% more active than SS-RJW100 in LRH-1 luciferase reporter assays. We present an LRH-1 crystal structure that illuminates striking mechanistic differences: SS-RJW100 adopts multiple configurations in the pocket and fails to make an interaction critical for activation by RR-RJW100. In molecular dynamics simulations, SS-RJW100 attenuates intramolecular signalling important for coregulator recruitment, consistent with previous observations that it weakly recruits coregulators in vitro. These studies provide a rationale for pursuing enantiomerically pure RJW100 derivatives: they establish RR-RJW100 as the stronger LRH-1 agonist and identify a potential for optimizing the SS-RJW100 scaffold for antagonist design.

## Introduction

Liver receptor homolog-1 (LRH-1) and steroidogenic factor-1 (SF-1) are closely related nuclear hormone receptors (NR) that are attractive drug targets due to key roles as regulators of metabolism, inflammation, and proliferation. The receptors share 53% sequence identity and recognize the same canonical DNA response element to drive transcription^1^. However, they are not functionally redundant due to discrete expression patterns^2–4^. LRH-1 is highly expressed in the liver, where it regulates lipid homeostasis and methyl metabolism^5–10^. In skeletal muscle, LRH-1 regulates glucose uptake^11^, and it controls local glucocorticoid synthesis in the intestinal epithelia^12,13^. Functions of SF-1 include regulation of energy homeostasis in the brain^14^, steroidogenesis in the ovaries and adrenal glands^4^, and sexual development^15^. LRH-1 modulators have therapeutic potential for nonalcoholic fatty liver disease, diabetes, inflammatory bowel diseases, and cancers^7,12,16–19^. SF-1 modulators are sought primarily for adrenocortical cancer^20,21^.

**Figure 1:**
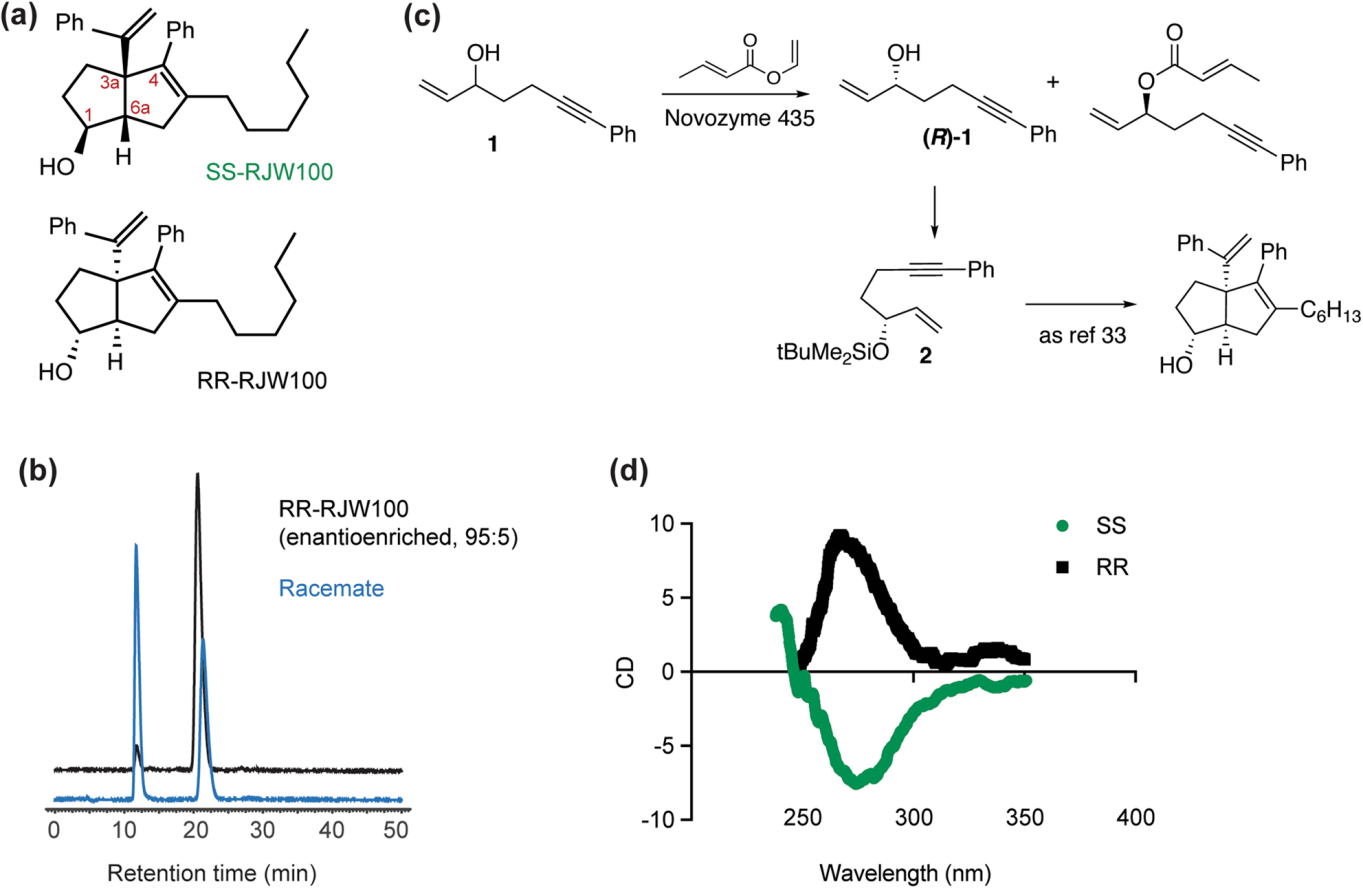
RJW100 enantiomers. (**a**) Chemical structures of SS- and RR-RJW100. (**b**) HPLC traces for RR-RJW100 generated by enantioselective synthesis (black curve) and for the racemate (blue curve). Separation by HPLC resulted in enantiopure RR-RJW100 and SS-RJW100 (both 100% ee). (**c**) Enantioselective synthesis of RR-RJW100. The (*R*)-enantiomer of the alcohol **1** was obtained from the racemate by kinetic resolution using a crotylation reaction with Novozyme 435, an immobilized *Candida antartica* lipase as the catalyst, which is known to be selective for the (S)-enantiomer of alcohols with a similar structure^54^. The reaction was stopped at around 50% conversion. The recovered enantioenriched alcohol (R)-**1** (95:5 enantiomer ratio) protected as the *tert*-butyldimethylsilyl ether and converted to RR-RJW100 (95:5 enantiomer ratio) using the published procedure for the racemate^33,34^. The enantio-enriched RR-RJW100 was used to identify the two HPLC peaks from the racemate and was not used for experiments with LRH-1/ SF-1. (**d**) CD traces of the RJW100 enantiomers used in these studies.

LRH-1 and SF-1 have very similar, lipophilic ligand binding pockets that have been challenging to target with synthetic molecules. The native ligands of these receptors are likely phospholipids (PL), as they have been crystallized with a variety of PL in the binding pockets. Unlike typical NR hormones, PL do not make polar interactions deep in the binding pocket, and they partially protrude into the solvent^22–27^. This unusual binding mode has made it difficult to predict types of synthetic molecules able to bind these receptors and modulate transcriptional activity. The compound RJW100 (Fig. 1a) is one of the few reported chemical scaffolds shown to activate these receptors and has been used by several groups to probe LRH-1 and SF-1 biology^16,27–33^.

RJW100 is a racemic mixture of two enantiomers that contain hexahydropentalene cores, two bulky styrene substituents, a hydrophobic *n-*hexyl substituent, and a hydroxyl group capable of making polar interactions (Fig. 1a). Differences in stereochemistry are found at positions 1 (the hydroxyl group), 3a (the external styrene), and 6a (Fig. 1a). Due to the cis-fused structure of the hexahydropentalene scaffold, positions 3a and 6a adopt the same absolute stereochemistry for RJW100: one enantiomer has *S* stereochemistry at both 3a and 6a, and the other has *R* stereochemistry in these positions. For simplicity, the RJW100 enantiomers will be hereafter called RR-RJW100 and SS-RJW100 for the stereochemistry at positions 1 and 3a/6a, respectively. Because of the bicyclic nature and differences in the stereochemistry, the hexahydropentalene cores of the enantiomers fold in opposite directions, thus resulting in very different three-dimensional structures.

The ability of each enantiomer to activate LRH-1 and SF-1 is unclear, as very few studies have evaluated them separately. Previous work has shown that both RR- and SS-RJW100 promote recruitment of coregulator protein fragments in vitro, a hallmark of ligand-driven NR activation or repression^34^. However, RR-RJW100 recruits 55% higher levels of the transcriptional intermediary factor 2 (Tif2) coactivator to LRH-1 than SS-RJW100 at saturation^34^. A greater capacity for recruiting coregulators could increase receptor transcriptional activity, but this has not been tested. Moreover, when LRH-1 is crystallized with racemic RJW100, the crystals unambiguously select for RR-RJW100^35,36^. Crystal selection for the same stereochemistry at position 3a is also seen for several RJW100 analogues^18,33,35^. The ability of certain enantiomers to promote crystallization may reflect biologically important conformational changes driven by ligand chirality. On the other hand, understanding how SS-RJW100 interacts in the pocket could inform new directions for structure-guided design.

Our laboratory has made significant progress in understanding the mechanism of action of RR-RJW100, leading to the discovery of compounds with improved potency and efficacy for LRH-1 and with selectivity for LRH-1 over SF-1^18,35–37^. However, we have not pursued enantiomerically pure RJW100 derivatives due to technical challenges associated with chiral separations. Considering the significant role of chirality in target recognition and pharmacology^38^ and to inform future drug design efforts, we investigated effects of the RJW100 enantiomers on LRH-1/ SF-1 activity and conformation. We report that the enantiomers have similar binding affinities for both receptors but have differing effects on receptor thermostability, suggesting distinct effects on protein conformation. We present the 1.7 Å crystal structure of LRH-1 bound to SS-RJW100, which shows that the ligand adopts two primary poses, both different from RR-RJW100 and related agonists. These binding modes are associated with weaker allosteric signalling to the site of coregulator association, reduced LRH-1 transcriptional activity in luciferase reporter assays, and an inability to make a hydrogen bond critical for activation of LRH-1 by RR-RJW100.

## Results

### Separation of RJW100 enantiomers

We synthesized RJW100 as a racemic mixture of two *exo* enantiomers, RR-RJW100 and SS-RJW100 (chemical structures shown in Fig. 1a). To separate the enantiomers from the racemate, we used a chiral preparative HPLC column, as previously described^34,35^. The enantiomers elute in two peaks with retention times of 11.7 min and 21.4 min (Fig. 1b). To determine the identity of the peaks, we compared the HPLC traces to a trace from RR-RJW100 prepared through an enantiomer-selective synthetic route, which yielded RR-RJW100:SS-RJW100 at a ratio of 95:5 (Fig. 1c)^34^. This HPLC analysis revealed that the slower-eluting peak contains RR-RJW100 (Fig. 1b). When analysed by circular dichroism, the two enantiomers produce peaks that are mirror images of each other, reflecting the differences in their chirality (Fig. 1d).

### Binding affinities of RJW100 enantiomers

**Figure 2:**
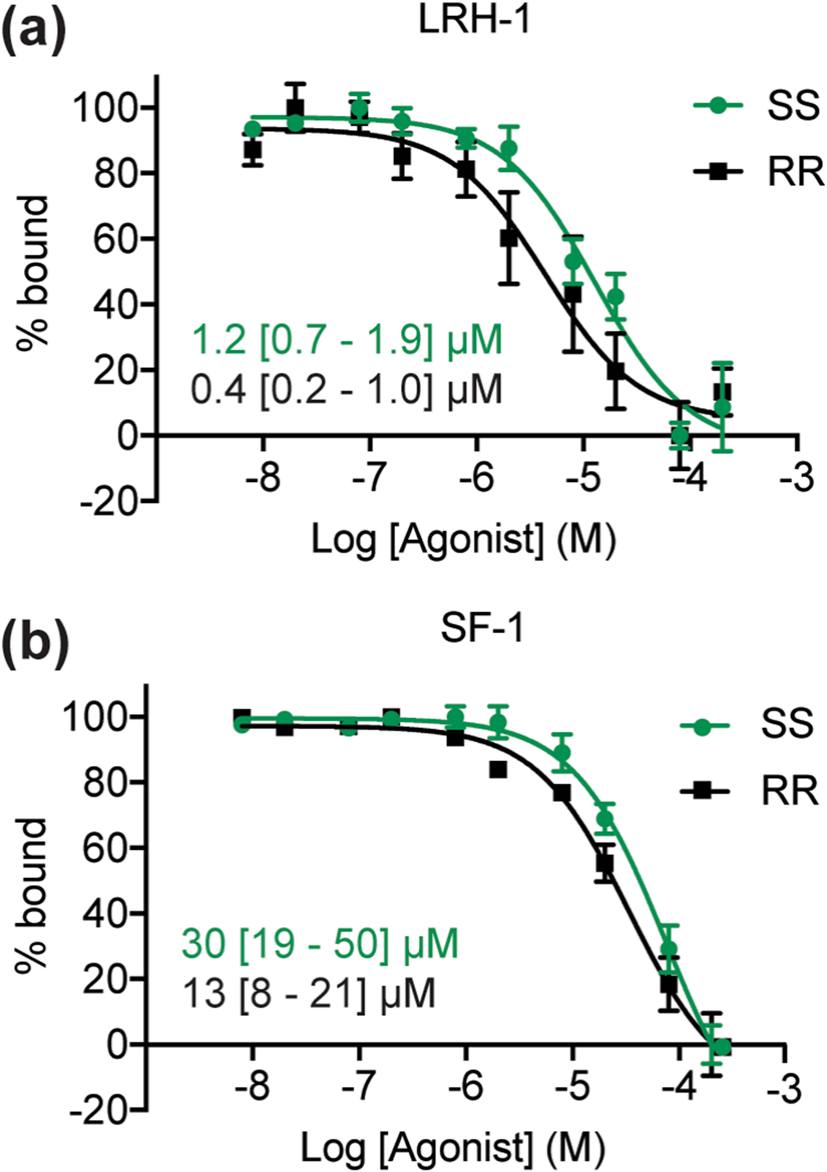
Binding affinities of RJW100 enantiomers. (**a**,**b**) Normalized FP dose–response curves from competition binding experiments using purified (**a**) LRH-1 or (**b**) SF-1 LDB showing that the ligands displace the probe. Each point represents the mean ± SEM of two independent experiments conducted in quadruplicate. Insets indicate the mean K_i_ values calculated from these curves (95% confidence intervals are in square brackets).

Previous coregulator recruitment studies indicate that both RJW100 enantiomers bind LRH-1 and SF-1^34^, but ligand binding affinities have not been measured. To determine binding affinities, we utilized a fluorescence polarization-based, equilibrium ligand binding assay in which a fluorescein-labelled probe is displaced by unlabelled competing ligands^39^. Differences in probe affinity for LRH-1 and SF-1 were calculated prior to the competition assays and are accounted for in the equation for fitting the competition data (details in the methods section and our previous publication)^39^. Both enantiomers outcompete the probe, indicating that they bind the receptors in the binding pockets (Fig. 2a,b). The RR-RJW100 enantiomer has an approximately threefold higher affinity for LRH-1 than SS-RJW100 (K_i_ = 0.4 µM versus 1.2 µM, Fig. 2a). Comparison of log K_i_ values calculated from three independent experiments show that the difference in affinity is statistically significant (p = 0.01 by two-tailed, paired t-test). However, magnitude of the affinity difference is fairly small and may not be biologically important. For SF-1, the binding affinity of RR-RJW100 trends higher than SS-RJW100, but the difference does not reach statistical significance (K_i_ = 13 µM for RR-RJW100 versus 30 µM for SS-RJW100, p = 0.2, Fig. 2b). Therefore, both enantiomers directly bind LRH-1 and SF-1 and do not exhibit dramatic differences in affinity.

### Differing effects of RJW100 enantiomers on receptor thermostability and transcriptional activity

**Figure 3:**
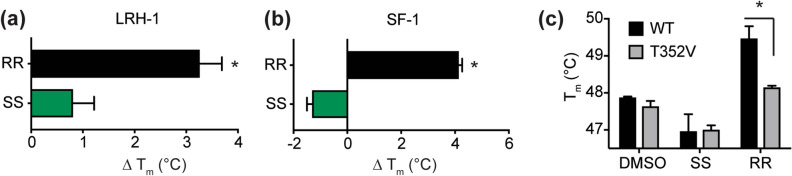
Enantiomer-specific effects on thermal stability of LRH-1 and SF-1. (**a**,**b**) Comparison of T_m_ values calculated from DSF experiments with (**a**) SF-1 or (**b**) LRH-1 LBD. Results are presented as the difference in T_m_ relative to protein bound to phospholipid. Significance of T_m_ differences was determined by two-tailed, paired Student’s t-tests from parallel experiments. *, p < 0.05. (**c**) DSF with wild-type (WT) or mutant (T352V) LRH-1 demonstrates the differential role of residue T352 in LRH-1 stabilization by RJW100 enantiomers. Each bar is the mean ± SEM for three experiments conducted in triplicate. Statistical significance was determined by two-way ANOVA followed by Sidak’s multiple comparisons test. *p < 0.05.

Although binding affinities are not dramatically different, the RJW100 enantiomers have distinct effects on protein stability and function. We used differential scanning fluorimetry (DSF) to probe the thermostability of purified receptor-ligand complexes, quantified by the temperature at which the complexes are 50% unfolded (T_m_). Proteins in solution are continuously sampling conformational states, and ligand-dependent changes in T_m_ provide insights into the energetics of receptor conformations recognized or induced by different ligands. Data are presented as change in T_m_ relative to PL-bound receptors. As previously observed^35^, RR-RJW100 stabilizes LRH-1 relative to a PL ligand (Fig. 3a). The RR enantiomer is similarly stabilizing to SF-1, increasing the T_m_ of the complex by around 4 °C (Fig. 3b). In contrast, SS-RJW100 does not significantly stabilize LRH-1 or SF-1 (Fig. 3a,b). Differences in T_m_ induced by the enantiomers are unrelated to differences in binding affinities, since DSF experiments utilized saturating ligand concentrations (20-fold molar excess). Mutagenesis experiments support the idea that these energetic differences arise from different ligand binding modes. One of the primary mechanisms used by RJW100 to bind and activate LRH-1 is a water-mediated hydrogen bond made by the ligand hydroxyl group with residue T352^35^, and we tested the effects of disrupting this interaction in DSF experiments. LRH-1 stabilization by RR-RJW100 is completely dependent on residue T352, but the T_m_ of the LRH-1-SS complex is unaffected by a T352V mutation (Fig. 3c). This indicates a differential reliance on the T352 interaction for binding LRH-1.

**Figure 4:**
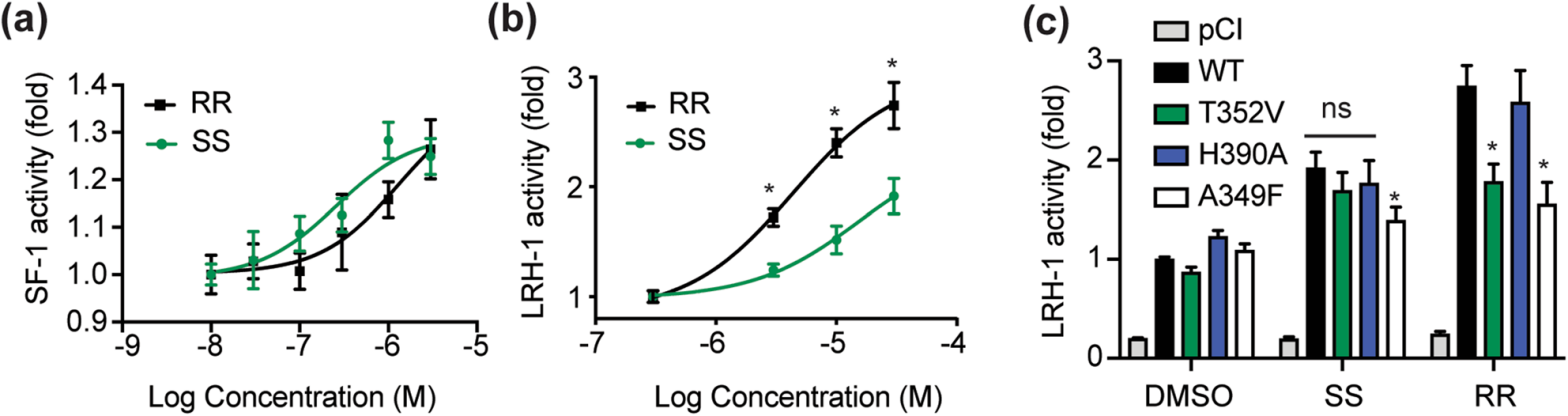
Effects on receptor transcriptional activity. (**a**,**b**). Luciferase reporter assays measuring transcriptional activity of full-length SF-1 (**a**) or LRH-1 (**b**) in Hela cells. Each point represents the mean fold change *versus* DMSO-treated cells ± SEM for three experiments each conducted in triplicate. Significance of differences in activity was determined by two-way ANOVA, followed by Sidak’s multiple comparisons test. *p < 0.05 *versus* SS-RJW100. (**c**) Mechanistic differences LRH-1 activation by SS-RJW100 compared to related molecules. Wild-type (WT) or mutant LRH-1 was overexpressed in Hela cells prior to treatment with 30 µM RR- or SS-RJW100. Each bar indicates mean fold change (± SEM) in LRH-1 activity after agonist treatment for 24 h relative to DMSO-treated cells. The experiment was conducted three times in triplicate. Fold change is calculated relative to DMSO-treated cells overexpressing WT LRH-1. Significance was determined by two-way ANOVA followed by Sidak’s multiple comparisons test. *p < 0.05 versus DMSO-treated cells for each group of bars (WT, mutant, or pCI empty vector. The A349F mutation introduces a bulky amino acid that blocks the binding pocket and was used as a negative control.

The T_m_ differences induced by the enantiomers suggest that they recognize different conformational states, which could impact receptor activation. Indeed, while both enantiomers similarly activate SF-1, RR-RJW100 is a much stronger activator of LRH-1 than SS-RJW100 in luciferase reporter assays (Fig. 4a,b). LRH-1 activation by SS-RJW100 is attenuated at all doses tested and is reduced by as much as 46% compared to RR-RJW100 (Fig. 4b). The weaker activity is associated with an inability to form a productive interaction with residue T352. Consistent with DSF studies (Fig. 3c) and our previous work^35^, RR-RJW100 is unable to activate a T352V LRH-1 mutant. However, this mutation does not affect activation by SS-RJW100 (Fig. 4c). SS-RJW100 activity also does not depend on an interaction with residue H390 like the closely-related analogue, GSK8470^40^. Our previous work demonstrated that GSK8470 cannot activate H390A-LRH-1^35^, but we find here that SS-RJW100-driven LRH-1 activity is unaffected by this mutation (Fig. 4c). Together, these results demonstrate that the chirality of RJW100 affects its ability to bind and activate LRH-1 but has a lesser impact on SF-1. Mutagenesis studies demonstrate that SS-RJW100 likely utilizes different mechanisms to activate the receptor than other agonists with the same core scaffold.

### Crystal Structure of SS-RJW100 bound to LRH-1

**Figure 5:**
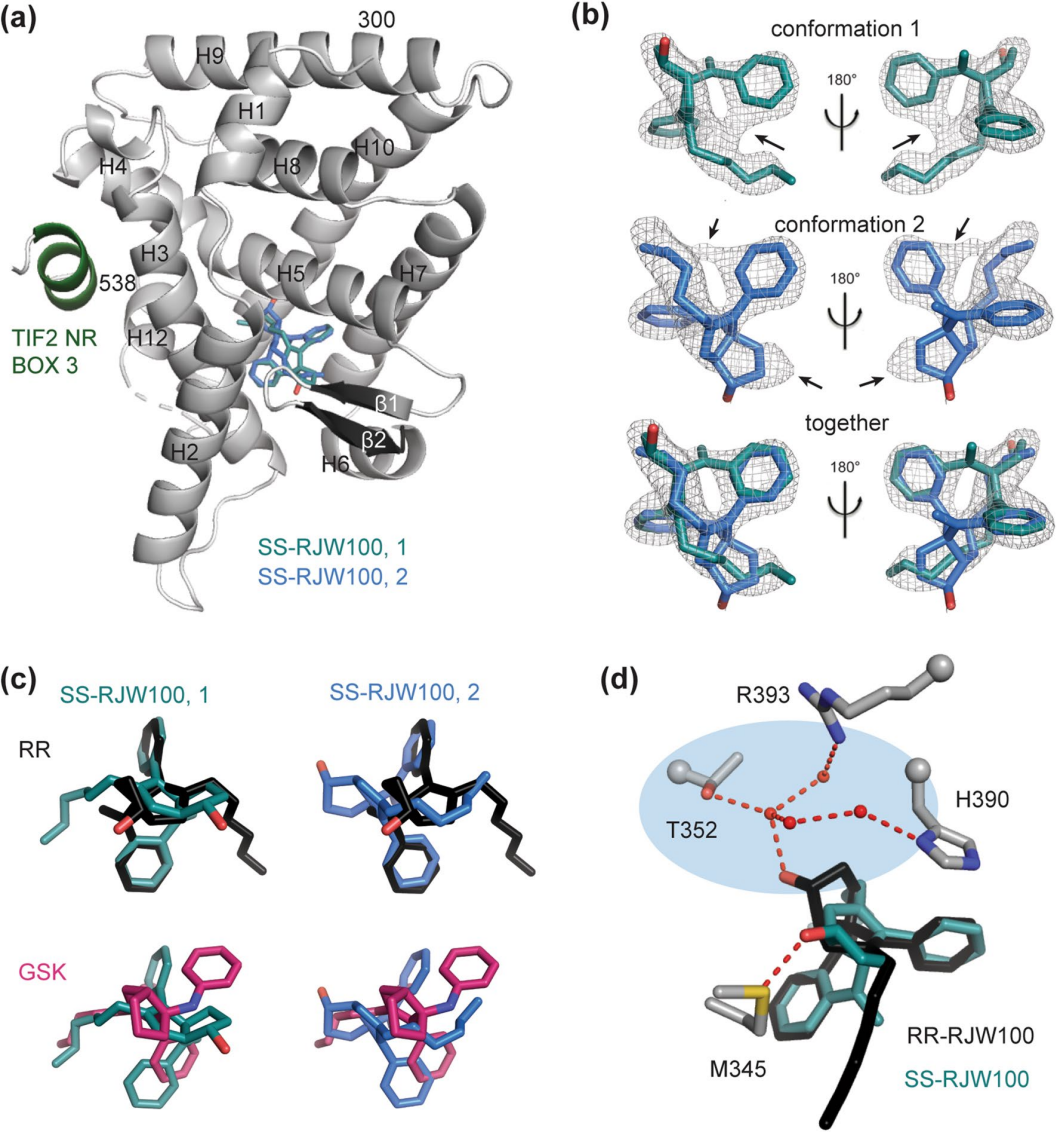
Crystal structure of LRH-1 bound to SS-RJW100. (**a**) Overall structure of the LRH-1 LBD (grey) bound to SS-RJW100 (two conformers, cyan and blue). The complex was crystallized with a fragment of the Transcriptional Intermediary Factor 2 (Tif2) coactivator, shown in *green*. H, helix. (**b**) Omit maps (F_O_-F_C_, contoured at 2 σ) showing the justification for modelling two orientations of the ligand. (**c**) Superpositions of SS-RJW100 with RR-RJW100 (*top*) and related molecule GSK8470 (*bottom*). (**d**) Different contacts made by SS-RJW100 and RR-RJW100 hydroxyl groups in the LRH-1 binding pocket. Highlighted in blue is the network of water molecules (red spheres) and a group of residues that they coordinate. Red dotted lines indicate hydrogen bonds. RR-RJW100, but not SS-RJW100 makes a water-meditated hydrogen bond with residue T352.

To understand how RR- and SS-RJW100 differentially affect LRH-1 activity, we determined the 1.7 Å X-ray crystal structure of SS-RJW100 bound to the LRH-1 ligand binding domain (LBD) (Table 1, Fig. 5a). The overall protein conformation is very similar to the published RR-RJW100-LRH-1 structure (PBD ID 5L11)^35^, with an average root mean square deviation (RMSD) of 0.1 Å. Strong electron density in the binding pocket indicates that the ligand is bound (Fig. 5b); however, modelling its precise position was challenging. Unlike in the RR-RJW100-LRH-1 structure, there is no clear indention in the density that indicates the puckering of the bicyclic core (see Supplementary Fig. S1 online). In addition, no single conformation of the ligand is sufficient to account for the electron density in the binding pocket (Fig. 5b). Modelling the ligand in two conformations with partial occupancies provides a better fit to the density and improves ligand B factors (values that reflect vibrational motion). With either one or two molecules in the model, B factors are higher for the ligand than for protein atoms in the structure, suggesting that the ligand is relatively mobile. However, the ratio of ligand/ protein B factors is 2.1 ± 0.4 with one molecule *versus* 1.63 ± 0.07 with two molecules modelled in the pocket, a significant improvement (p = 6 × 10^–12^ by two-tailed, unpaired Student’s t-test). Refinement of the structure indicates that the two SS-RJW100 conformers are present with nearly equal occupancies (52% occupancy for conformer 1 and 48% occupancy for conformer 2).

**Table 1:** X-ray data collection and refinement statistics.

**Data collection**	LRH-1/SS*-*RJW100 /Tif2
Space group	P4_3_2_1_2
**Cell dimensions**	
*a*, *b*, *c* (Å)	46.6 46.6, 220.9
α,β,γ (°)	90, 90, 90
Resolution (Å)	50–1.70 (1.76–1.70)*
*R*_pim_	0.04 (0.29)
*I*/σ*I*	18.2 (1.75)
CC_1/2_	(0.788)
Completeness (%)	97.4 (84.9)
Redundancy	3.8 (2.4)
**Refinement**	
Resolution (Å)	1.70
No. reflections	48,242
*R*_work_/*R*_free_ (%)	21.1 / 22.8
**No. atoms**	
Protein	2041
Water	106
**B-factors**	
Protein	37.7
Ligand	61.5
Water	43.3
**R.m.s. deviations**	
Bond lengths (Å)	0.003
Bond angles (°)	0.571
Ramachandran favoured (%)	99
Ramachandran outliers (%)	0
PDB accession code	6VC2

*Values in parenthesis indicate highest resolution shell.

The binding modes of the two SS-RJW100 molecules are very different than observed for RR-RJW100 and for the related agonist GSK8470 (seen through superposition of the structure with PDB 5L11 and PDB 3PLZ, respectively) (Fig. 5c). The two SS-RJW100 molecules are also positioned very differently from each other: the hydroxyl groups point in opposite directions (Fig. 5c). The ligand makes several hydrophobic interactions (see Supplementary Fig. S1 online), but neither orientation places the hydroxyl group near the water network coordinating residue T352 like RR-RJW100 (Fig. 5d). This explains why T352V mutation does not affect LRH-1 activation by SS-RJW100 (Fig. 4c). Instead, the SS-RJW100 hydroxyl group is within hydrogen bonding distance of LRH-1 residue M345 (conformation 1, Fig. 5d) or the backbone carbonyl of S383 (conformation 2, see Supplementary Fig. S1 online). However, the complete absence of density surrounding the hydroxyl groups for both conformers (Fig. 5b) suggests that these interactions are unstable.

In contrast to the hydroxyl group, the ligand density is very strong surrounding the phenyl rings from the external and internal styrene moieties (positions 3a and 4, respectively, Fig. 1a). Interestingly, the phenyl rings of the two conformers overlap despite the differences in the overall orientations of the molecules and the different sizes of the groups at positions 3a and 4 (Fig. 5b). They also overlap precisely with the phenyl rings from RR-RJW100 (Fig. 5c). This observation implies an important role for the styrene moieties in orienting the agonist in the pocket that is maintained even when it results in a nonideal positioning of the ligand hydroxyl group.

### The “frustrated” SS-RJW100 ligand fails to form stable interactions in the LRH-1 binding pocket

**Figure 6:**
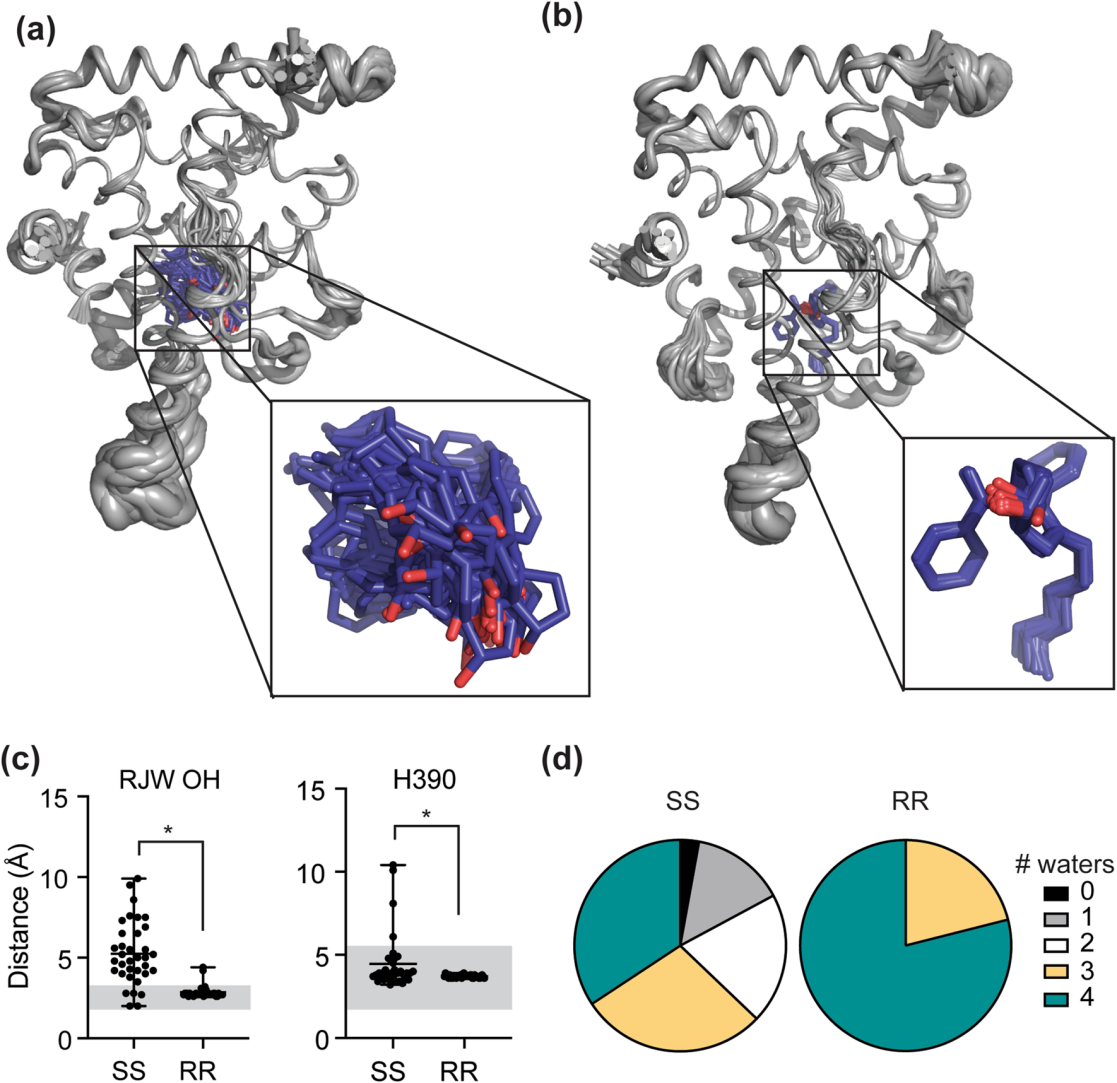
Modelling structural mobility with ensemble refinement. (**a**,**b**) Models of LRH-1 bound to either SS-RJW100 (**a**) or RR-RJW100 (**b**) generated through Ensemble Refinement, resulting in 35 coordinate models for the SS-RJW100-LRH-1 structure and 38 for LRH-1-RR-RJW100 (superposed in the figure). The input models for ensemble refinement were PDB 6VC2 (from this publication) with one conformation of SS-RJW100 modelled in the pocket and PDB 5L11, respectively^35^. The ensembles are represented as cartoon “putty,” in which the size of the cartoon is proportional to residue B factors. *Insets* depict close-up views of the ligand conformations in each model of the ensemble. (**c**) Left, Stability of hydrogen bonds made by the RJW100 hydroxyl groups was determined by measuring the distances between the hydroxyl groups and the closest binding interaction in each model of the ensembles. For SS-RJW100, we measured the distance between OH and M345 (sulphur atom), and for RR-RJW100 we measured the distance between the OH and the water atom coordinating residue T352. Grey shaded area indicates distances in hydrogen-bonding range. *Right*, Stability of π-π stacking with residue H390 was measured in each frame. Grey shaded area indicates distances in bonding range. (**d**) The intact water network contains four molecules, but this network is disrupted in the SS-RJW100 ensemble. The pie chart summarizes the number of water molecules in each model in the ensemble.

Because of the difficulty placing SS-RJW100 in the structure, we sought to improve our model using Ensemble Refinement, a tool in the Phenix software suite^41^. This tool refines crystallographic data using a combination of molecular dynamics simulations and translation-libration-screw (TLS) to model local vibrational disorder and global disorder, respectively. This generates an ensemble of models that fits the crystallographic data better than a single model^41^. Superposition of the 35 models in the ensemble shows more motion in solvent-exposed regions, particularly near the bottom of the molecule, while the internal part of the structure is more stable (Fig. 6a). This is not surprising and is very similar to an LRH-1-RR-RJW100 model generated through ensemble refinement (Fig. 6b). However, there is a dramatic difference with the ligands: SS-RJW100 adopts many very different conformations within the binding pocket in the composite model, while RR-RJW100 exhibits a single preferred conformation (Fig. 6a,b, inset). As a result, there is a marked difference in the stability of the hydrogen bond made by the hydroxyl group of each enantiomer (with LRH-1 residue M345 for SS-RJW100 or with the water molecule coordinating residue T352 for RR-RJW100). SS-RJW100 makes an OH-mediated hydrogen bond in only five out of the 35 models (14%), while RR-RJW100 maintains the hydrogen bond in 95% of the models (p < 0.0001, by two-tailed, unpaired Student’s t-test Fig. 6c, left). Interestingly, the interaction made by the SS-RJW100 styrene with LRH-1 residue H390 is much more stable: edge-to-face π-π stacking occurs in 88% of the models (Fig. 6c, right). However, this is less stable than the same interaction by RR-RJW100, which persists in 100% of the models (Fig. 6d, p = 0.009 by two-tailed, unpaired Student’s t-test). Finally, SS-RJW100 has a destabilizing effect on the water network that coordinates a small group of polar residues in the LRH-1 binding pocket (including T352). This network contains four water molecules, and destabilization of the network is associated with lower activity of LRH-1 agonists^35^. In the LRH-SS-RJW100 ensemble, the water network is intact for only 35% of the models versus 79% of the models when the RR enantiomer is bound (Fig. 6d).

To explore ligand dynamics more rigorously, we conducted 1 microsecond molecular dynamics simulations (MDS) with the enantiomer-bound LRH-1 structures. All trajectories had stable RMSDs of less than 1.9 Å (see Supplementary Fig. S2 online). Over the course of the simulation, hydrogen bonding by the RR-RJW100 hydroxyl group with the water molecule coordinating T352 is maintained for 58.2% of the time. Hydrogen bonding of SS-RJW100 with either M345 (with the ligand starting in conformation 1) or S383 (starting in conformation 2) is less stable, persisting for 16.2% and 23.7% of the simulations, respectively. We also measured the stability of edge-to-face π–π stacking with H390, defined as having an angle between the ring planes between 60 and 120° and distance within 5.5 Å between rings^35,42^. The interaction is maintained for 62.0% of the simulation with RR-RJW100, 56.1% of the time for SS-RJW100 (conformer 1), and 48.7% of the time for SS-RJW100 (conformer 2). These results are consistent with the analyses from Ensemble Refinement. Therefore, the reduced activity of SS-RJW100 is associated with an inability to form stable, directional interactions in the binding pocket.

### SS-RJW100 diminishes LRH-1 allosteric activation networks

**Figure 7:**
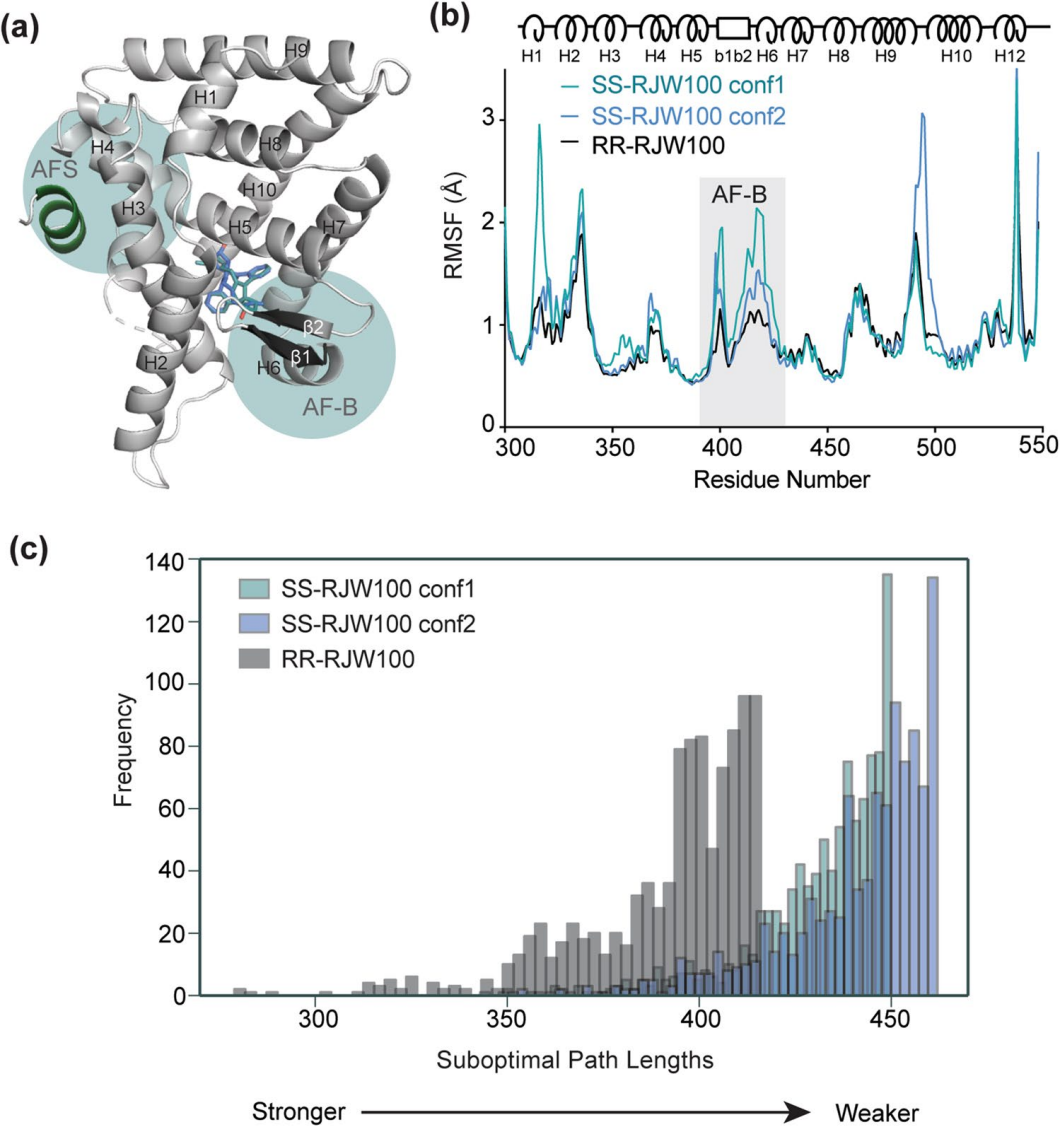
Weaker allosteric signalling by SS-RJW100 in MDS. (**a**) Model of the LRH-1 LBD with the AF-B and AFS highlighted in cyan. (**b**) RMSFs for each complex over the course of the 1 microsecond simulation. The diagram at the top of the graph indicates the position in the protein. Grey box indicates the AF-B. (**c**) Histogram of the top 1000 suboptimal paths between AF-B and the AFS for the three complexes.

Activation of LRH-1 by ligands involves an allosteric signalling network between the ligand binding pocket and the activation function surface (AFS), the site of coregulator binding. A key part of this allosteric network is the helix 6/ β-sheet surface (AF-B) near the ligand binding pocket (Fig. 7a). The AF-B is utilized by both synthetic and phospholipid agonists to modulate coregulator associations via allosteric communication with the AFS in a mechanism involving correlated motion between these two distant sites^22,24,36^. To understand how two ligands with similar binding affinities differentially affect LRH-1 transcriptional activity, we investigated whether chirality affects receptor conformation and allostery in MDS. First, we examined ligand-dependent differences in receptor conformation that might impact allosteric signalling. A comparison of root mean square fluctuation (RMSF) of Cα atoms in each complex over the course of the simulations shows generally higher fluctuations in the presence of either SS-RJW100 conformer *versus* RR-RJW100 (Fig. 7B). This is consistent with the lower thermostability of the LRH-1-SS-RJW100 complex described in Fig. 3. Notably, the AF-B is the most prominent region destabilized by both SS-RJW100 conformers (Fig. 7b). Since the AF-B to AFS communication axis involves correlated motion between these two sites, the marked difference in RMSF in AF-B suggests the potential that this communication is altered when SS-RJW100 is bound.

To quantify the allosteric communication between the AF-B and AFS in the LRH-1-RJW100 complexes, we constructed dynamical networks and conducted suboptimal path analysis. Communication between distant sites in a protein occurs through correlated motion of residues along paths between them. These paths of communication can follow thousands of possible routes, but the paths containing the strongest correlated motion (the optimal path and a subset of suboptimal paths) convey the most information. In suboptimal path analysis, each Cα is considered a “node,” and the nodes are connected to each other by “edges.” The length of an edge is inversely proportional to the correlated motion of the two nodes it connects, such that shorter edges indicate more correlation^24,43^. The sum of edge lengths along a path therefore indicates the strength of the path. A histogram comparing the lengths of the 1000 shortest suboptimal paths between the AF-B and AFS for the three complexes reveals that stronger communication occurs when RR-RJW100 is bound compared to either conformer of SS-RJW100 (Fig. 7c). This evidence of stronger communication to the site of coregulator binding is consistent with the higher E_max_ of Tif2 binding to LRH-1 in the presence of RR-RJW100 previously reported^34^. Reduced ligand-driven communication to the AFS provides an explanation for the lower levels of transcriptional activity induced by the SS enantiomer (Fig. 4c) and its reduced ability to recruit the Tif2 coactivator^34^.

### Potential of the SS-RJW100 binding pose for antagonist design

**Figure 8:**
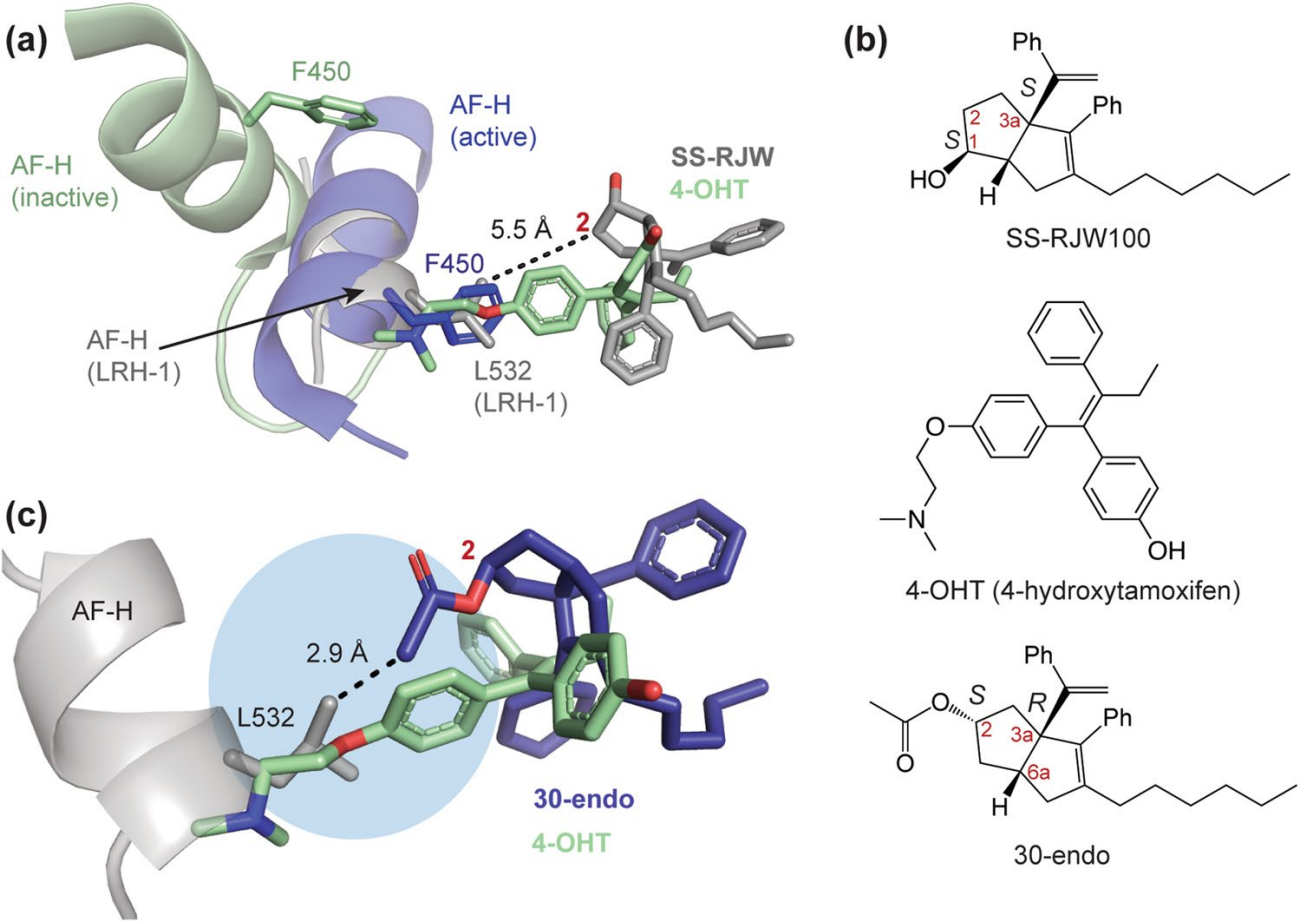
Potential for exploiting the SS-RJW100 binding mode to generate LRH-1 antagonists. (**a**) Superposition the ligands (sticks) and AF-H (cartoon) from three structures: oestrogen receptor gamma in the active state (dark blue, from PBD 2GP7), bound to 4-hydroxy tamoxifen (4-OHT, pale green, from PDB 2GPU)^45^, and LRH-1-SS-RJW100 (grey). The clash of 4-OHT with residue L532 is shown. (**b**) A comparison of chemical structures of SS-RJW100, 4-OHT, and 30-endo (**c**) Docking of the compound 30-endo (dark blue sticks)^33^ in the SS-RJW100 structure as an example of how bulky groups could be added to the SS-RJW100 scaffold to extend toward the AF-H. The acetoxy moiety appended to the ligand bicyclic core at position 2 is within 3 Å of the AF-H and nearly overlaps with TAM. The blue circle indicates space that could accommodate bulk and displace the activation function helix (AF-H) in a similar manner to TAM.

Design of LRH-1 antagonists has been particularly challenging, and we sought to gain insights from the SS-RJW100 binding mode that could aid future antagonist design. Nuclear receptor antagonists often disrupt the (AFS) through physical displacement of the activation function helix (AF-H), thereby interfering with coregulator recruitment^44^. A classic example of this is 4-hydroxytamoxifen (4-OHT), an antagonist of the oestrogen receptor and oestrogen related receptors (Fig. 8a,b). The 4-OHT mechanism of action is illustrated in Fig. 8a, which shows the superposition of two structures of oestrogen related receptor gamma (ERRγ) in the active state or bound to 4-OHT (from PBD 2GP7 and 2GPU, respectively)^45^. The clash of 4-OHT with residue F450 of ERRγ causes displacement of the AF-H (Fig. 8a). To explore the potential for the SS-RJW100 scaffold as an antagonist, we examined its proximity to the LRH-1 AF-H. Both dominant binding modes of SS-RJW100 orient it such that portions of the molecule are within 6 Å of residue L532 on the LRH-1 AF-H, which is in the analogous position to ERRγ F450. Conformer 2 is slightly closer, with position 2 of the molecule 5.5 Å from L532 (Fig. 8a). Therefore, the addition of bulk at position 2 could result in AF-H displacement. To illustrate this point, we docked a previously-described RJW100 analogue named “30-endo”^33^ into the LRH-1-SS-RJW100 structure, using the enantiomer with the same ring pucker as SS-RJW100 (Fig. 8b,c). The acetoxy group at position 2 of 30-endo follows a trajectory toward the AF-H and is 2.9 Å away from L532 (Fig. 8c). Incorporating additional bulk into the group at position 2 could increase this clash and may result in an effective LRH-1 antagonist.

## Discussion

Chirality is important for drug design: one enantiomer is often responsible for on-target activity, while the other could have no effect, on-target beneficial effects, or deleterious effects^38^. In these studies, we uncovered striking functional differences between the RJW100 enantiomers. Compared to RR-RJW100, SS-RJW100 destabilizes LRH-1 and is a weaker activator in luciferase reporter assays (Figs. 2, 3, 4). The reduced activity is associated with unstable hydrogen bonding by the ligand and impaired allosteric signalling between the AF-B and the AFS (Figs. 5, 6, 7). These findings provide a rationale for pursuing enantiomerically pure versions of RJW100 and its derivatives, suggesting that improved LRH-1 activity could be achieved with R stereochemistry at the 3a position for molecules with this scaffold. Curiously, the differences between the enantiomers are much less pronounced for SF-1 than LRH-1. SS-RJW100 destabilizes SF-1 relative to RR-RJW100 in DSF assays, but it does not significantly affect binding affinity or transcriptional activity. The reason that SF-1 is less sensitive is unclear. SF-1 has a very similar binding pocket to LRH-1, with 53% sequence identity overall and 61% sequence identity in the LBD. Understanding the reason SF-1 is less sensitive than LRH-1 to RJW100 chirality will likely require a structure of SF-1 bound to a synthetic molecule, which is not currently available.

In addition to delineating mechanisms that cause differential activity of the RJW100 enantiomers, these studies provide insights that could guide future drug design. Development of LRH-1/ SF-1 modulators has been challenging, in part because natural ligands of these receptors (PL) interact with the receptors in an unusual manner compared to many other NR ligands^22–27^. The capacity for the deep part of the LRH-1/ SF-1 pockets to accommodate synthetic ligands is not fully understood nor apparent from observing PL-bound structures. Structural and mechanistic studies with small molecule agonists have established the importance of two deep pocket interactions in positioning ligands with the same 3a stereochemistry as RR-RJW100. The first is the hydrogen bond with a small polar patch in the deep binding pocket near residue T352, and the second is π-π stacking with residue H390^18,33,35,36^. With RR-RJW100, the configuration of the π-π stacking and hydrogen-bonding sites is complementary to the shape of the pocket, allowing an extremely stable binding mode. Understanding this has been vital for LRH-1 agonist development, enabling the discovery of potent and efficacious new lead agonists with a robust structure–activity relationship^18,37,39^. Likewise, the novel binding poses of SS-RJW100 present opportunities to target other regions of the binding pocket. This could be especially important for development of LRH-1 antagonists, which has lagged behind agonist discovery. There are no published crystal structures of LRH-1 bound to an antagonist, and the current leading antagonist binds with similar affinity to SS-RJW100^39,46^. Notably, both conformations of SS-RJW100 in the structure prevent an interaction with T352, a critical driver of LRH-1 activation. This property is favourable for an antagonist, particularly in combination with other modifications that improve affinity or enhance receptor inactivation. While the instability of SS-RJW100 may be viewed as an impediment to rational design, several factors highlight the potential of this stereochemistry. The strong electron density surrounding the styrene rings, together with dynamical analyses, suggest these regions are stable relative to the rest of the molecule and are likely driving positioning of the ligand (Figs. 5, 6, 7). Stabilizing a desired conformation of the ligand could be possible through modifications to molecule that increase binding site compatibility. For example, one could envision adding bulk to the bicyclic core to stabilize conformation 2 and dispalce the AF-H, a common strategy in NR antagonist design (Fig. 8).

Together, these studies have illuminated key differences in the function of RJW100 enantiomers. Insights gained from this work further the understanding of how LRH-1 and SF-1 are regulated by synthetic ligands and will aid future endeavours in drug discovery for these receptors.

## Materials and methods

### Chemical synthesis

Racemic RJW100 was synthesized as previously described^33,34^. The enantiomers were separated from the racemate on a 4.6 × 250 mm Daicel OD-H HPLC column run at 1 ml/min in 1% isopropanol in hexane. This gives SS-RJW100 and RR-RJW100 as enantiopure materials:

- (1*S*,5*S,*6*S*)-3-Hexyl-6-hydroxy-2-phenyl-1-(phenylvinyl)-bicyclo[3.3.0]oct-2-ene (SS-RJW100): R_t_ 11.7 min on chiral HPLC on a Diacel OD-H, (1 mL/min 1% isopropanol in hexane on 4.6 × 250 mm column).
  [α]_D_^25^ –46.7 (c = 0.48, CHCl_3_, 100% e.e.).
- (1*R*,5*R,*6*R*)-3-Hexyl-6-hydroxy-2-phenyl-1-(phenylvinyl)-bicyclo[3.3.0]oct-2-ene (RR-RJW100): R_t_ 21.4 min on chiral HPLC on a Diacel OD-H, (1 mL/min 1% isopropanol in hexane on 4.6 × 250 mm column).
  [α]_D_^25^ + 45.1 (c = 0.49, CHCl_3_, 100% e.e.).

The identity of the individual enantiomers was established though an enantioselective synthesis of *RR-*RJW100 from (*R*)-(7-phenylhept-1-en-6-yn-3-yloxy)(tert-butyl)dimethylsilane (also see Fig. 1c):

- (*R*)*-*(7-Phenylhept-1-en-6-yn-3-yloxy)(*tert*-butyl)dimethylsilane ((*R*)*-*2): A solution of racemic 7-phenylhept-1-en-6-yn-3-ol (0.372 g, 2.00 mmol) in vinyl crotonate (0.91 mL) was placed in Schlenk tube and an immobilized *Candida antartica* lipase (Novozyme 435) was added (0.091 g). The reaction was incubated at 42 °C (without stirring) for 72 h. After this time, the reaction mixture was filtered through sintered funnel, and the resins were washed with dry Et_2_O. The filtrate was concentrated *in vacuo* to give the crude products as a yellow oil. Purification by column chromatography on SiO_2_ with hexane:ethyl acetate (4:1) as the eluent gave (*R*)-7-phenylhept-1-en-6-yn-3-ol **1** as a pale yellow oil (0.179 g, 96%, 95:5 enantiomer ratio) and (*E*)-(*S*)-7-phenylhept-1-en-6-yn-3-ylbut-2-enoate as a yellow oil (0.229 g, 90%).
- Synthesis of RR-RJW100: To a stirred solution of (*R*)-7-phenylhept-1-en-6-yn-3-ol **1** (0.179 g, 0.96 mmol) in dry dichloromethane (12.0 mL) was added dropwise Et_3_N (0.39 mL, 2.82 mmol) at 0 °C. The stirring was continued at the same temperature for 5 min before dropwise addition of ^t^BuMe_2_SiOTf (0.33 mL, 1.41 mmol). After stirring for 10 min at 0 °C the ice bath was removed, and the stirring continued for 30 min at RT before quenching with H_2_O (20 mL). The whole mixture was poured onto H_2_O (100 mL) and the products extracted with dichloromethane (2 × 75 mL). The combined organic phases were washed with H_2_O (2 × 100 mL) and brine (100 mL), dried over MgSO_4_, filtered and concentrated *in vacuo* to give the crude material as a yellow oil. Purification by flash chromatography column on Al_2_O_3_ (basic, grade III) with hexane as the eluent provided (*R*)-(7-phenylhept-1-en-6-yn-3-yloxy)(tert-butyl)dimethylsilane ((*R*)-**2**) as a pale yellow oil (0.193 g, 67%) whose spectral data was consistent with those obtained for racemic product^33^. Chiral HPLC showed that the compound so formed corresponded to the slower running of the two enantiomers and retained the 95:5 enantiomeric ratio of the starting enyne (Fig. 1b).

### Circular dichroism

100 µM solutions of SS- or RR-RJW100 were prepared from 10 mM DMSO stocks by diluting 1:100 in deionized water. Solutions were sonicated for ~ 20 s in a bath sonicator. Sonicated samples were added to a cuvette (3 mm path length) and read on a Jasco J-810 spectropolarimeter. Samples were scanned through wavelengths of 700–190 nm at a flow rate of 200 ml/min.

### Protein purification

LRH-1 ligand binding domain (LBD, residues 299–541) in the pMSC7 vector was transformed in *E. coli* strain BL21(pLysS) and grown in Liquid Broth (LB) to an optical density (OD_600_) of 0.6. Protein expression was induced with 1 mM isopropyl-1-thio-D-galactopyranoside (IPTG) for four hours at 30 °C. Protein was purified by nickel affinity chromatography, using 20 mM Tris–HCl pH 7.4, 150 mM NaCl, 5% glycerol, and either 25 mM imidazole (Buffer A) or 500 mM imidazole (Buffer B). Protein used for ligand binding assays and DSF was incubated with dilauroylphosphatidylcholine (DLPC, Avanti Polar Lipids; Alabaster, AL) at fivefold molar excess, overnight at 4 °C. Following DLPC exchange, protein was re-purified by size exclusion chromatography into assay buffer (150 mM NaCl, 20 mM Tris–HCl (pH 7.4), and 5% glycerol). Mutant LRH-1 (T352V) was purified using the same conditions as wild-type LRH-1. SF-1 LBD (residues 218–461) in the pMSC7 vector was transformed in *E. Coli* strain BL21 (pLysS) and grown in LB at 37 °C to an OD_600_ of 0.6. Expression was induced with 0.5 mM IPTG, followed by overnight growth at 18 °C. SF-1 was purified as described above for LRH-1 except for the addition of 5 mM tris(2-carboxyethyl)phosphine (TCEP) to the nickel affinity chromatography buffers and that it was not exchanged with DLPC prior to re-purification by size exclusion chromatography. The vector for His-tagged Tobacco Etch Virus (TEV) protease was a gift from John Tesmer (University of Texas at Austin). The pMSC7 vector was provided by John Sondek (University of North Carolina at Chapel Hill).

### Mutagenesis

Point mutations to LRH-1 in the pCI vector or in the pMSC7 vector have been described previously^35^.

### Fluorescence polarization (FP) binding assay

Assays utilized a fluorescein-labelled probe (6 N-FAM) that is displaced by unlabelled competitors^39^. Competition assays were conducted in opaque, black 384 assay plates using a combination of 10 nM 6 N-FAM with 5 nM LRH-1 ligand binding domain (LBD) or 30 nM 6 N-FAM with 25 nM SF-1 LBD (these concentrations were determined through optimization steps in our previous work)^39^. Competitor ligand concentrations ranged from 2^–11^ to 2^–4^ M. A constant amount of DMSO was present in each well (6.7% v/v). Assays were conducted three times in quadruplicate. FP was measured on a BioTek Neo plate reader. GraphPad Prism (v.8) was used to analyze the data using a one-site, fit K_i_ curve, which fits the K_i_ of the unlabelled competing ligand using the EC_50_ of the competition curve, the concentration of the fluorescent probe, and the K_d_ of the probe for each receptor, as follows:

Equation 1: Calculating the EC_50_ of the competing ligand.

1 $${\rm Y} = {\rm Bottom} + {\rm (Top{-}Bottom)}/(1 + 10^\wedge  ({{X - {\rm LogEC}_{{50}} }} )) $$

Equation 2: Calculating K_i_ from EC_50_, probe concentration, and probe K_d_.

2 $${\text{logEC}}_{{50}}  = {\rm{log}}(10^\wedge {{{\rm{log}}{\text{K}}_{\text{i}} }} *(1 + [\rm Probe,nM]/Probe\;K_d,nM)). $$

Significance of differences in K_i_ values from parallel experiments was determined using two-tailed, paired t-tests, with p < 0.05 considered statistically significant.

### Differential scanning fluorimetry

Purified protein (0.2 mg/ ml, in an assay buffer of 20 mM Tris HCl pH 7.5, 150 mM NaCl, and 5% glycerol) was combined with RJW100 enantiomers (100 µM) overnight at 4 °C. SYPRO orange dye (Thermo Fisher) was added to the complexes the next day, at a final dilution of 1:1000. Complexes were heated at a rate of 0.5 °C/min on a StepOne Plus thermocycler (Applied Biosystems), with excitation/ emissions wavelengths of 488 nm/603 nm. Background was subtracted from reactions containing agonists and dye but no protein. Three replicates were performed in triplicate, using two separate protein preparations. T_m_ was calculated using the Bolzman equation (GraphPad Prism, v.8). Significance of differences in T_m_ shifts were determined using two-tailed, paired t-tests, with p < 0.05 being considered statistically significant.

### Cell culture

Hela cells were purchased from and authenticated by Atlantic Type Culture Collection. Cells were cultured in phenol red-free MEMα medium (Thermo Fisher) supplemented with 10% charcoal-stripped foetal bovine serum. Cells were maintained under standard culture conditions.

### Reporter gene assays

Hela cells were plated in white-walled, clear-bottomed 96-well plates. Cells were seeded at densities of 7500 cells/ well, yielding a confluency of ~ 70–80%. The following day, cells were transfected with the FugeneHD transfection reagent (Promega) at ratios of 5 µl Fugene: 2 µg DNA. Cells in each well were transfected with 5 ng full-length LRH-1 in a pCI vector (wild-type or with point mutations as described in the figure legends) and two reporter plasmids: (1) containing a portion of the SHP promoter cloned upstream of firefly luciferase in the pGL3 basic vector (20 ng/well) and (2) a constitutive *Renilla* luciferase vector under the control of the CMV promoter, used as a control for transfection efficiency (1 ng/well). Cells transfected with the pCI empty vector and reporter plasmids were used as negative controls. Cells were treated with agonists 24 h after transfection at concentrations indicated in the figure legends. The DualGlo kit (Promega) was used to quantify luminescence signal 24 h after treatment on a BioTek Neo plate reader. SF-1 luciferase experiments were conducted identically, except that full-length SF-1 (in a pcDNA3.1 vector) was overexpressed instead of LRH-1 (with empty pcDNA3.1 as the negative control).

### Crystallography

LRH-1 LBD was purified by nickel affinity chromatography as described above. The His-tag was cleaved from its fusion partner using tobacco etch virus (TEV) protease overnight with dialysis at 4 °C into imidazole-free buffer A. Cleaved protein was incubated with SS-RJW100 overnight at 4 °C (fivefold molar excess). The protein–ligand complex was re-purified by size exclusion into a buffer of 100 mM ammonium acetate (pH 7.5) 100 mM sodium chloride, 1 mM EDTA, 2 mM DTT, and 2 mM 3-[(3-cholamidopropyl)dimethylammonio]-1-propanesulfonate (CHAPS). Following size exclusion chromatography, additional compound was added to ensure saturation (fivefold molar excess) and combined with a peptide corresponding to the NR Box 3 of the Tif2 coactivator with the sequence H_3_N- KENALLRYLLDKDDT-CO_2_ (RS Synthesis). This complex was concentrated to 6 mg/ml and used for crystallization. Crystals were generated by hanging drop vapor diffusion at 18 °C, using microseeding to aid nucleation. Seeds were derived from crystals of racemic RJW100 bound to LRH-1 (generated as previously described)^35^. Drops contained protein:crystallant:seeds at a 1:1:1 ratio. The crystallant was 0.05 M sodium acetate, pH 4.6 and 5–11% PEG4000.

### Crystal data collection and structure refinement

Crystals were flash-frozen in liquid nitrogen, using a cryoprotectant of crystallant in 30% v/v glycerol. Data were collected remotely at the Argonne National Laboratory, South East Regional Collaborative Access Team, Beamline 22ID. Data were processed with HKL2000^47^ and phased by molecular replacement in Phenix^48^, using PBD 5L11 as the search model. Ensemble refinement was performed using the Phenix program “Ensemble Refine”^41^. Phenix version 1.16–3549 was used with the Ensemble Refine package: https://www.phenix-online.org/.

### Molecular dynamics simulations

All molecular dynamics simulations were performed for 1000 ns with Amber18^49^ as previously described^50^. Three models of the LRH-1 ligand binding domain were used: (1) with RR-RJW100 (PDB 5L11), (2) with SS-RJW100 (conformer 1 from PBD 6VC2), or (3) with SS-RJW100 (conformer 2 from PDB 6VC2). Complexes were solvated in an octahedral box of TIP3P water with 10 Å buffer supplemented with Na^+^ and Cl^-^ ions at 150 mM. Dynamic Network analyses were carried out by selecting Cα atoms in the protein as nodes for network construction. A pair of nodes was connected by edges if they maintained a distance of < 4.5 Å for at least 75% of the simulation. Carma and NetworkView plugin in Visual Molecular Dynamics (VMD)^51^ were utilized for producing the dynamic network^52^. The edge distances were derived from pairwise correlations as a measure of communication within the network. Suboptimal paths between the AF-B and the AFS sites were identified using the Floyd-Warshall algorithm^53^ and analysed by the subopt program in the VMD NetworkView plugin^51^. Cα atoms of residues 410 and 534 were used as source and sink nodes.

### Molecular modelling

An enantiomer of the “30-endo” ligand ((2*S*,3a*R*,6a*S*)-5-hexyl-6-phenyl-6a-(1-phenylvinyl)-1,2,3,3a,4,6a-hexahydropentalen-2-yl acetate)^33^ was modelled into the SS-RJW100-LRH-1 structure using Maestro v. 12.1.013 (Schrodinger) by editing conformation 2 of SS-RJW100 and performing energy minimization.

## Supplementary Information

Supplementary Legends.

Supplementary Information 2.

Supplementary Information 3.

Supplementary Information 4.

Supplementary Information 5.

## Data Availability

Coordinates and structure factors have been deposited in the Protein Data Bank with the accession numbers, 6VC2.
