# Supplementary material for: Enantiomer-specific activities of an LRH-1 and SF-1 dual agonist
Source: Sci Rep. 2020 Dec 17;10:22279. doi: 10.1038/s41598-020-79251-9 (PMC7747700; doi:10.1038/s41598-020-79251-9)
Supplement: Supplementary file 1 — Supplementary Legends. [file 41598_2020_79251_MOESM1_ESM.docx]

**Enantiomer-specific activities of an LRH-1 and SF-1 dual agonist**

Suzanne G. Mays^1^, Józef Stec^2^, Xu Liu^1^, Emma H. D’Agostino^1^, Richard J. Whitby^2^, and Eric A. Ortlund^1*^

^1^ Emory University, Department of Biochemistry, Atlanta, GA, 30322, United States

^2^ University of Southampton, School of Chemistry, Southampton, Hants, SO17, United Kingdom

*eortlun@emory.edu

Supplemental Figure Legends

**Figure S1.** Comparison of LRH-1 structures bound to either SS-RJW100 or RR-RJW100. A. Superposition of the omit electron density maps from the ligand binding pockets of structures of LRH-1 bound to either RR-RJW100 (PBD 5L11, purple mesh) or SS-RJW100 (PBD 6VC2, grey mesh). Clear differences are evident between the shapes of the maps. The RR-RJW100 density has an indentation in the density (black arrow) indicating the pucker of the bicyclic core, while the position of the core is more ambiguous for SS-RJW100. Omit electron density shown is 2Fo-Fc, contoured at 2.5 sigma. B-C. Contacts made by SS-RJW100 in the two modelled conformations. Amino acids shown as grey sticks all make contacts with the ligand. *Black dotted line* indicates pi-pi stacking. *Red dotted line* indicates a hydrogen bond. All other residues shown make hydrophobic contacts with the ligand.

**Figure S2.** Quality control in MDS. Plot of root mean square deviations (RMSDs) over time for each complex in molecular dynamics simulations. Values are stable over the 1000 ns simulation.
