# Supplementary figures and images for: Enantiomer-specific activities of an LRH-1 and SF-1 dual agonist
Source: Sci Rep. 2020 Dec 17;10:22279. doi: 10.1038/s41598-020-79251-9 (PMC7747700; doi:10.1038/s41598-020-79251-9)

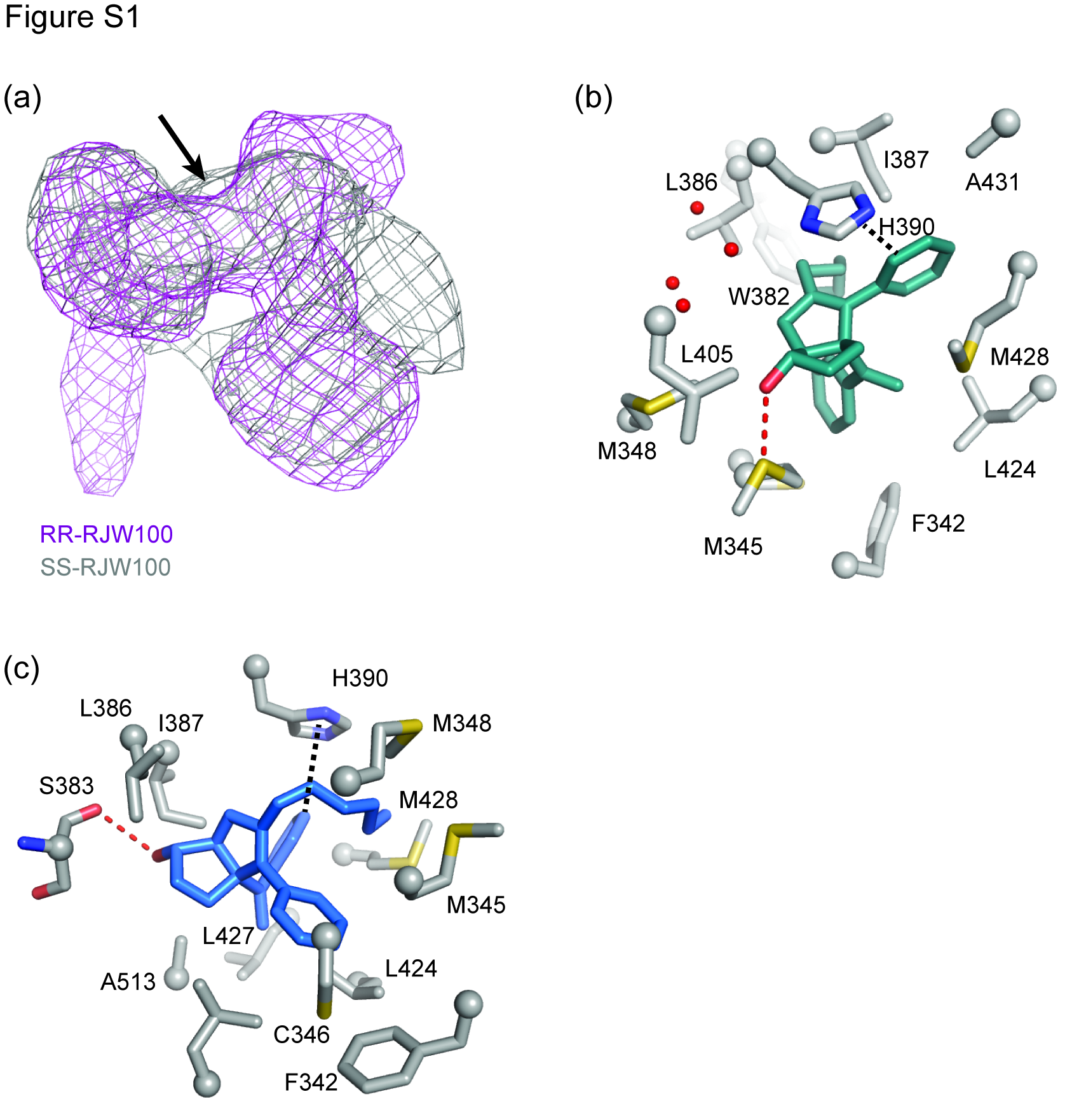

Supplement: Supplementary file 2 — Supplementary Information 2. [file 41598_2020_79251_MOESM2_ESM.tif]

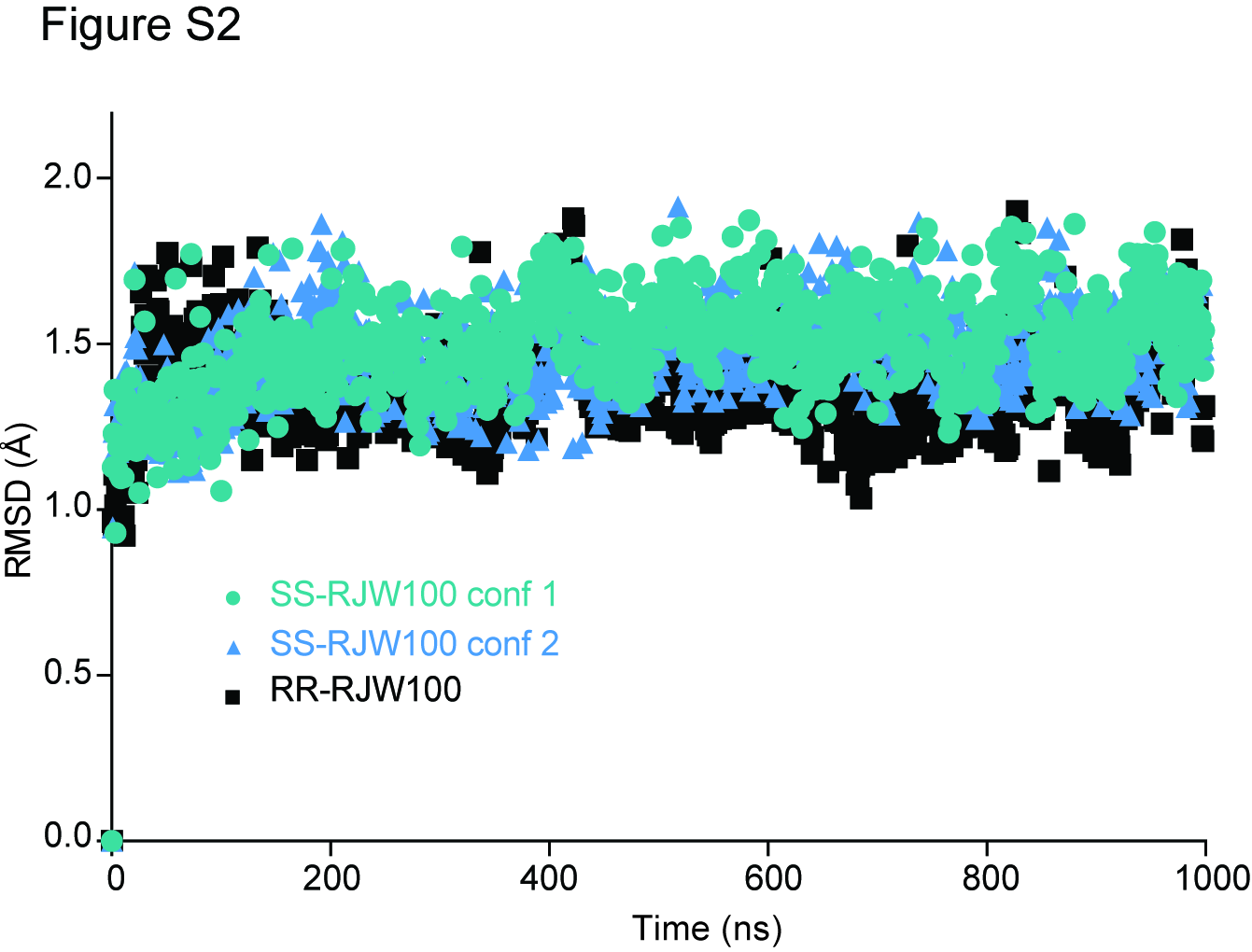

Supplement: Supplementary file 3 — Supplementary Information 3. [file 41598_2020_79251_MOESM3_ESM.tif]
