# Supplementary material for: Enantiomer-specific activities of an LRH-1 and SF-1 dual agonist
Source: Sci Rep. 2020 Dec 17;10:22279. doi: 10.1038/s41598-020-79251-9 (PMC7747700; doi:10.1038/s41598-020-79251-9)
Supplement: Supplementary file 4 — Supplementary Information 4. [file 41598_2020_79251_MOESM4_ESM.pdf]

Data File E:\04_10001.D
Sample Name: js/5271/16c1a

===

Acq. Operator	: rjw	Seq. Line :	1
Acq. Instrument	: LC 1	Location :	Vial 11
Injection Date	: 04/08/2008 20:55:31	Inj :	1
		Inj Volume :	3.000 µl

Acq. Method : C:\HPCHEM\1\METHODS\1IPAHEXL.M
Last changed : 04/08/2008 20:54:24 by rjw
Analysis Method : C:\CHEM32\2\METHODS\DEF_LC.M
Last changed : 15/11/2018 10:57:44 by Richard Whitby

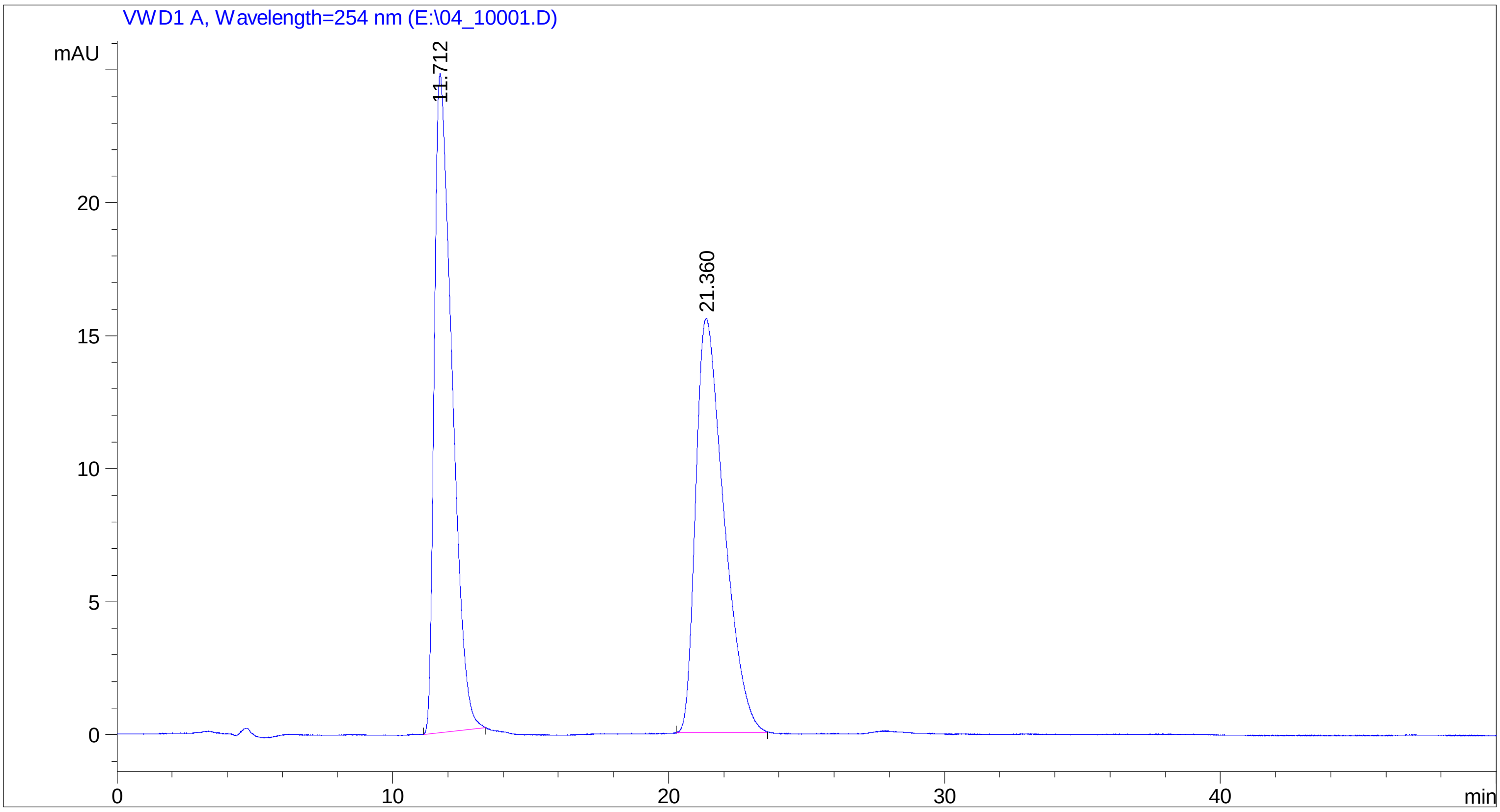

===
Area Percent Report
===

Sorted By : Signal
Multiplier: : 1.0000
Dilution: : 1.0000
Use Multiplier & Dilution Factor with ISTDs

Signal 1: VWD1 A, Wavelength=254 nm

Peak #	RetTime [min]	Type	Width [min]	Area [mAU*s]	Height [mAU]	Area %
1	11.712	BB	0.5323	1104.59607	24.77033	49.8572
2	21.360	BV	0.8366	1110.92236	15.58478	50.1428

Totals : 2215.51843 40.35511

===
*** End of Report ***
