# Supplementary material for: Enantiomer-specific activities of an LRH-1 and SF-1 dual agonist
Source: Sci Rep. 2020 Dec 17;10:22279. doi: 10.1038/s41598-020-79251-9 (PMC7747700; doi:10.1038/s41598-020-79251-9)
Supplement: Supplementary file 5 — Supplementary Information 5. [file 41598_2020_79251_MOESM5_ESM.pdf]

Data File E:\04_10002.D
Sample Name: js/5271/19c

=====================================================================

Acq. Operator : rjw
Seq. Line : 2
Acq. Instrument : LC 1
Location : Vial 12
Injection Date : 04/08/2008 21:46:07
Inj : 1
Inj Volume : 3.000 µl
Acq. Method : C:\HPCHEM\1\METHODS\1IPAHEXL.M
Last changed : 04/08/2008 20:54:24 by rjw
Analysis Method : C:\CHEM32\2\METHODS\DEF_LC.M
Last changed : 15/11/2018 10:57:44 by Richard Whitby

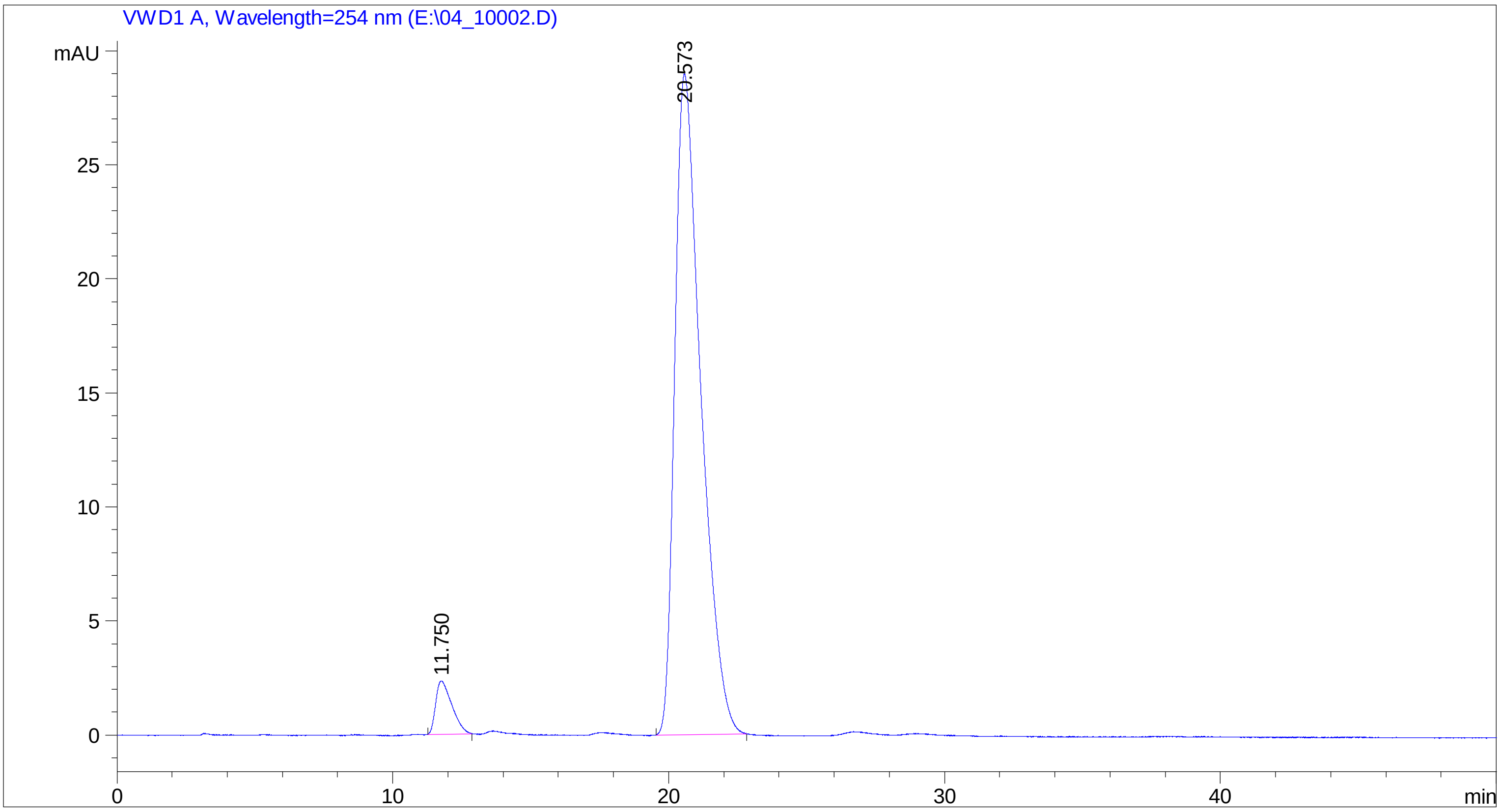


=====================================================================

Area Percent Report

=====================================================================

Sorted By : Signal
Multiplier: : 1.0000
Dilution: : 1.0000
Use Multiplier & Dilution Factor with ISTDs

Signal 1: VWD1 A, Wavelength=254 nm

| Peak # | RetTime [min] | Type | Width [min] | Area [mAU*s] | Height [mAU] | Area % |
|---|---|---|---|---|---|---|
| 1 | 11.750 | BB | 0.4812 | 94.58358 | 2.33683 | 4.7249 |
| 2 | 20.573 | BB | 0.7719 | 1907.22595 | 28.97673 | 95.2751 |

Totals : 2001.80953 31.31356

=====================================================================

*** End of Report ***
